# Supplementary material for: CRISPR/Cas9‐mediated somatic correction of a novel coagulator factor IX gene mutation ameliorates hemophilia in mouse
Source: EMBO Mol Med. 2016 Mar 10;8(5):477–88. doi: 10.15252/emmm.201506039 (PMC5125832; doi:10.15252/emmm.201506039)
Supplement: Supplementary file 1 — Appendix [file EMMM-8-477-s001.pdf]

CRISPR/Cas9 mediated somatic correction of a novel coagulator factor IX gene mutation ameliorates hemophilia in mouse

Supplementary Information List

Table of Contents

Appendix Figures 5
Appendix Tables 6

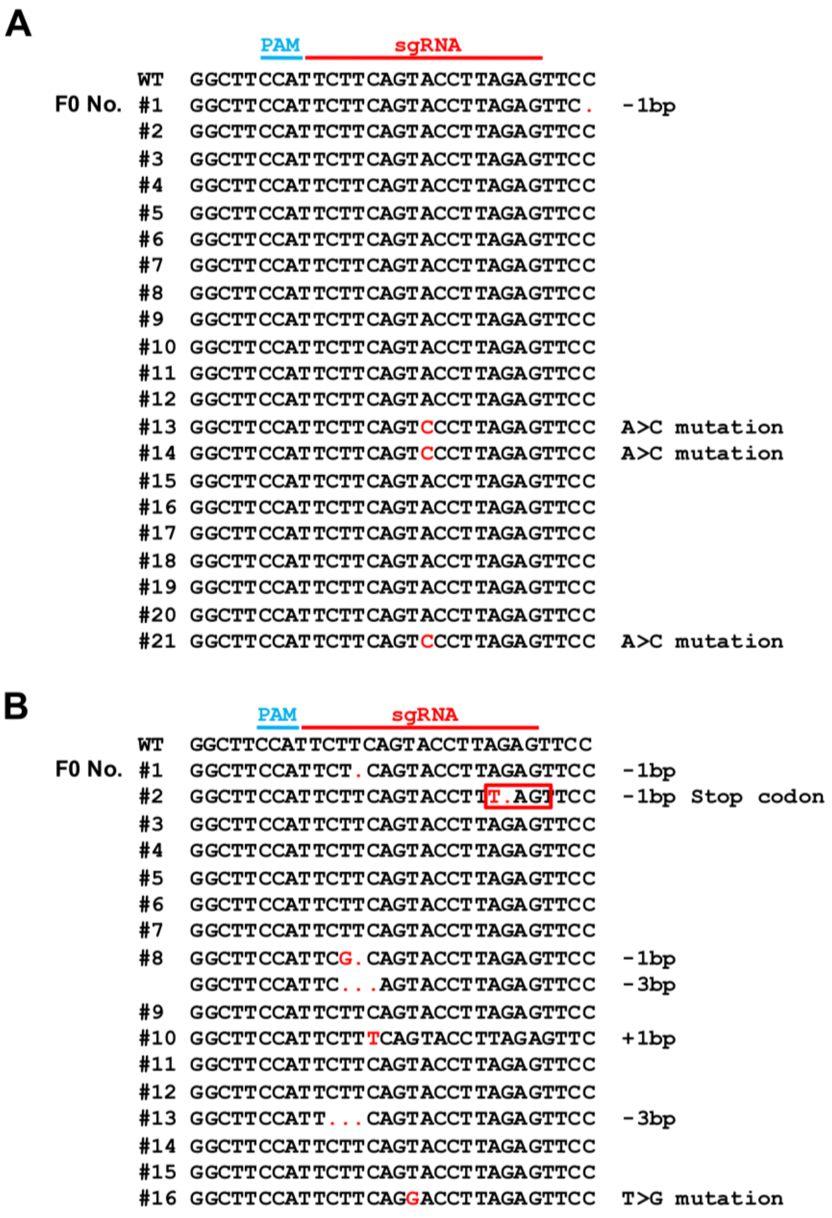

Appendix Figure S1. Generation of variant mouse strains with *F9* mutation via Cas9-mediated genome editing.

A The founder sequences of $F9^{Y381S}$ mouse strain are shown. Nucleotide substitutions were indicated by a red letter. **B** The founder sequences of $F9^{Y381D}$ and $F9^{382Stop}$ strains are shown. Nucleotide substitutions were indicated by a red letter and deletions by dots; premature stop codon is boxed in red.

A

	sequence	score	UCSC gene	locus
OT1	CCTTTCTTCAGAACCATAGAGAT	1.4	NM_001164818	chr2:-156393557
OT2	CTTTTCTTCAGTACCTGAGTTTA	0.9	NM_007882	chr18:-20146875
OT3	CTCTTCTTCAGGACCTGAAAGTT	0.9	NM_001033460	chr5:+30657485
OT4	CTTCTCTTCAGTACCATATGGTT	0.7	NM_177184	chr9:-67795908
OT5	CTCTTCTTCAGTAAGTAAGGGTT	0.5	NM_175089	chr8:+63578702
OT6	CTCTTCTTCAGAACCTGATATTT	0.5	NM_001080924	chr11:-5238542
OT7	CCGTTCTTCAGAACCTTTGGGCT	0.4	NR_102726	chr1:-173531247
OT8	CTGTCCTTCAGCACCTTAAAGGT	0.3	NM_025281	chr5:+38625321
OT9	CCATTCTTCACTACATCAGAATT	0.2	NM_027823	chr9:+8995857
OT10	CTATTCATCAGAACCTTGGATTT	0.2	NM_194055	chr4:+11294015

B

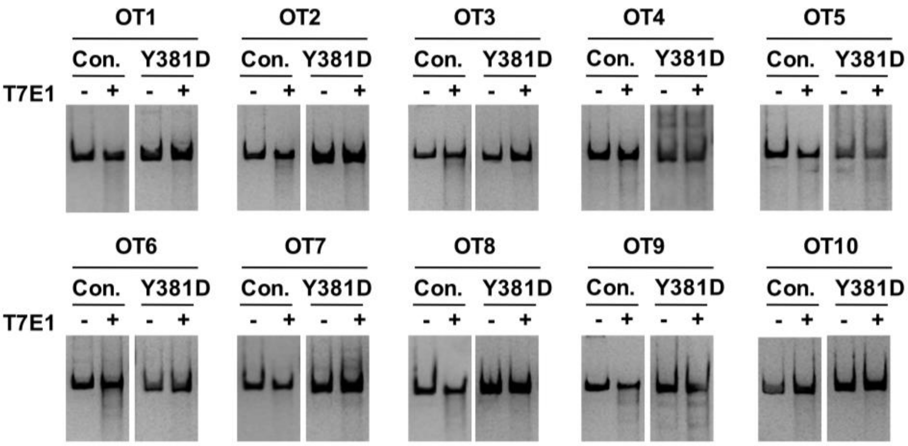

Appendix Figure S2. Off-target analysis of the sgRNA used for generation of *F9* mutant mice.

A The 10 most likely potential off-target sites predicted by Optimized CRISPR Design website (http://crispr.mit.edu/) are listed with their sequence, score, gene ID and genomic locus. **B** T7E1 assays were performed with PCR products from the founder mouse of the $F9^{Y381D}$ strain. No off-target mutation was detected by sequencing of 50 TA-clones of each PCR product.

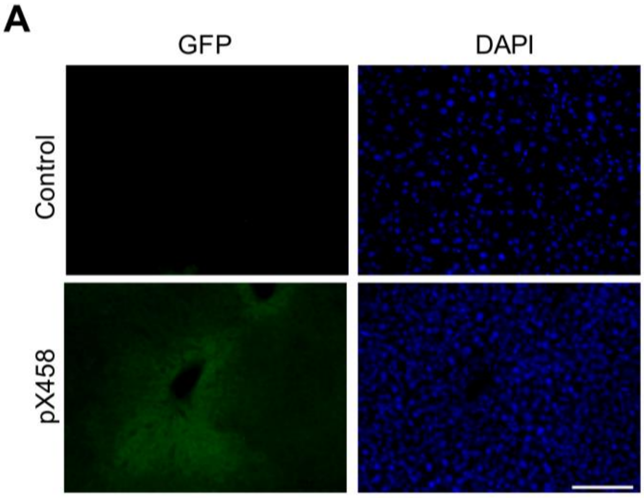

ROIs	Positive	Total	Ratio
1	228	929	24.5%
2	341	1136	30.0%
3	210	1022	20.5%
4	360	1607	22.4%
5	39	942	4.1%
6	105	987	10.6%
7	270	1185	22.8%
8	111	1052	10.6%
Average	208	1107.5	18.2%

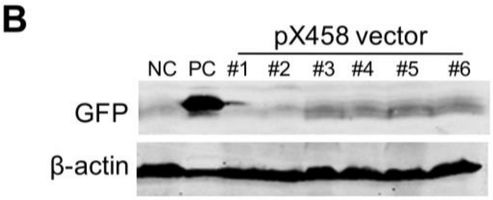

Appendix Figure S3. Expression of Cas9-2A-GFP protein in liver tissue from mice 20h after hydrodynamic injection of 120 μg pX458 construct.

A Frozen sections were prepared from mouse liver tissues with or without plasmid DNA treatment. Upper panel: the sections were strained with 2-(4-Amidinophenyl)-6-indolecarbamidine dihydrochloride (DAPI) and fluorescence images were taken with a microscope. Scale bar: 100μm. Lower panel: list of individual region of interests (ROIs). In each ROI, the GFP positive and total cell numbers were counted and the average percentage of GFP signals was calculated. **B** GFP expression was detected through western blotting with antibodies against GFP. β-actin was used as an internal loading control. NC: negative control without plasmid injection; PC: positive control of 293T cells transfected with pX458. The other lanes are individual mice that received pX458 through hydrodynamic tail vein injection.

A

	sequence	score	UCSC gene	locus
OT1	CCATTCTTCAGTACCTTAGAGTT	49.2	NM_007979	chrX:-57282327
OT2	CTCTTCTTCAGGACCTGAAAGTT	3.7	NM_001033460	chr5:+30657485
OT3	CCTTTCTTCAGAACCATAGAGAT	1.4	NM_001164818	chr2:-156393557
OT4	CTCTTCTTCAGGACCTCAAAGAC	0.9	NM_011086	chr1:-65301691
OT5	CTTTTCTTCAGGGCCTTAAAATA	0.8	NM_013930	chr6:-23024686
OT6	CCATTCTTCATGAGCTCAGAGTT	0.6	NM_001165944	chr10:-129403721
OT7	CCCTTCTTCATGAGCTTAGGGTG	0.5	NM_011439	chr1:+135279172
OT8	CCATTCTTCAGGCCCATGGAGTG	0.5	NM_182745	chr5:+107974825
OT9	CTCTTCTTCAGAACCTGATATTT	0.5	NM_001080924	chr11:-5238542
OT10	CCGTTCTTCAGAACCTTTGGGCT	0.4	NR_102726	chr1:-173531247

B

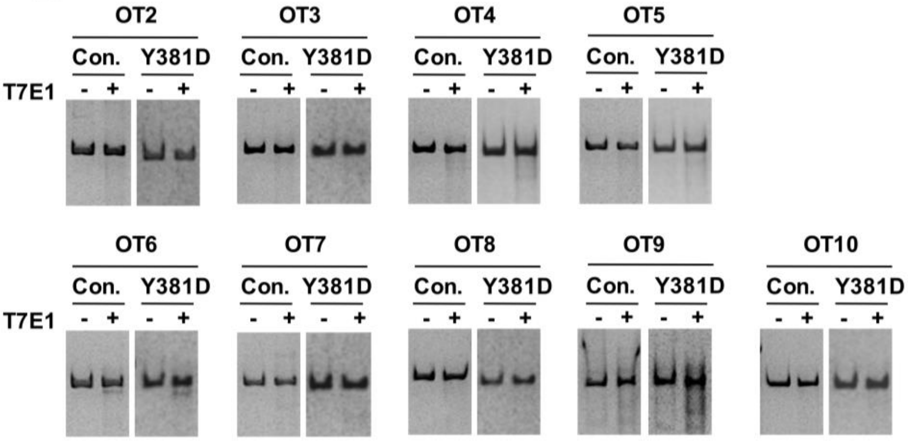

Appendix Figure S4. **Off-target analysis of the sgRNA used for correction of the $F9^{Y381D}$ mutation in mice.**

A The 10 most likely potential off-target sites predicted by Optimized CRISPR Design website (http://crispr.mit.edu/) software are listed with their sequence, score, gene ID and genomic locus.**B** T7E1 assays were performed with PCR products amplified from DNA extracted from liver tissues of the mice treated with 1×10^{10} and 7×10^{10} vector genomes of AdvCas9 and AdvG/T, respectively. No off-target mutation was detected by sequencing of 50 TA-clones of each of 9 PCR products.

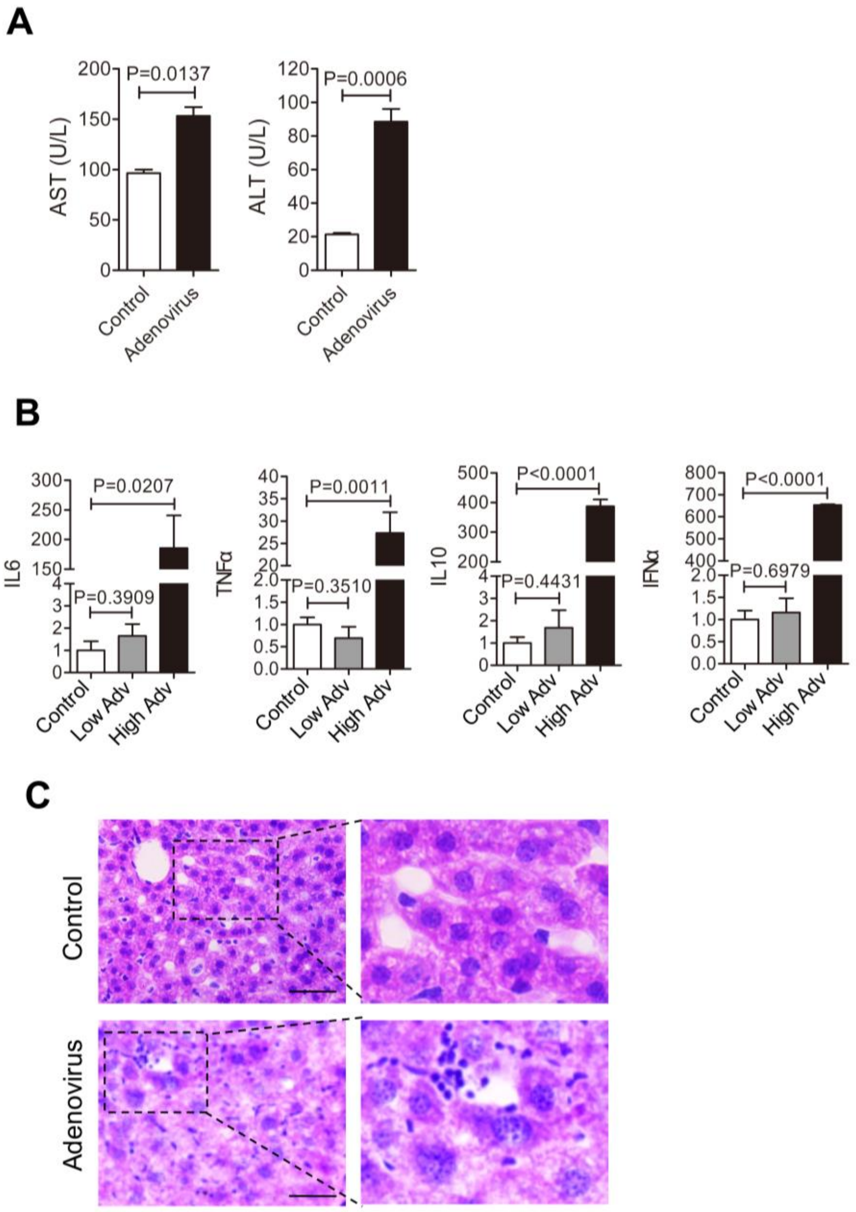

Appendix Figure S5. Liver damage was evaluated through molecular and histological studies.

A Aspartate aminotransferase (AST) and alanine aminotransferase (ALT) were tested in peripheral blood from F9^{Y381D} mice treated with or without 1×10^{10} and 7×10^{10} vector genomes of AdvCas9 and AdvG/T for 8 weeks (n=5). **B** mRNA levels of inflammatory cytokines were determined by qRT-PCR. mRNA samples were harvested in control or AdV treated mice described in (A). **C** Typical H&E staining images of liver tissues from mice treated with or without AdVs. Scale bar=100μm.

Appendix Table S1 sgRNA sequences.

ID	Sequence (5'-3')
F9^{WT}	CTCTAAGGTACTGAAGAA
F9^{Y381D}	AACTCTAAGGTCCTGAAGAA

Appendix Table S2 ssODN sequences.

ID	Sequence (5'-3')
F9 T>G	TGGCTGGGGAAAAGTCTTCAACAAAGGGAGACAGGCTT<br>CCATTCTTCAG**G**ACCTTAGAGTTCCACTGGTGGATAGAG<br>CCACATGCCTTAGGTCCACAAC
F9 A>C	GGCTGGGGAAAAGTCTTCAACAAAGGGAGACAGGCTTC<br>CATTCTTCAGT**C**CCTTAGAGTTCCACTGGTGGATAGAGCC<br>ACATGCCTTAGGTCCACAACA
F9^{Y381D} G>T	TGTCAGTGGCTGGGGAAAAGTCTTCAACAAAGGGAGAC<br>AGGCTTCCATTCTTCAG**T**ACCTTAGAGTTCCACTGGTGGA<br>TAGAGCCACATGCCTTAGGTCCACAACATTCACTATCTAT<br>AA

Appendix Table S3 primer sequences for off target analysis of sgRNA for generation of F9^{Y381D} mutant mice.

ID	Sequence (5'-3')
F9-OT1-F	GGTGACAAGGAAGATGATGGG
F9-OT1-R	CAGAAGGACTCCAGAGTACAGC
F9-OT2-F	CCATTAGCTCTTCAGTCGTGG
F9-OT2-R	CTCTAGTGTCTGGTACACTGATGC
F9-OT3-F	CAGAGCTGGGCTATGCATGTATAG
F9-OT3-R	CTGCCGTATCTGCTTGGTGTAT
F9-OT4-F	GTCTGTGTACCATATGCATGCC
F9-OT4-R	AGCAAGTTGTCCTACACCTCCC
F9-OT5-F	GTTGCAACAATCAGAGTTGGGG
F9-OT5-R	GGGCCTTTTGGGATAGCATT
F9-OT6-F	GGCCCCAAAGATGTAAGGTACAG
F9-OT6-R	TCTTAGATGCACCCACTAGGCCT
F9-OT7-F	GTCTGCTCATCCGCTGGTTCTA
F9-OT7-R	CTCTGACTTCAAACTCAGGAGCC
F9-OT8-F	CTGAGGCAGCTGCTTATCTTC
F9-OT8-R	AACAACACCCCAACCTCATC

F9-OT9-F	CCTTGACCAAGGAGCTTAGCA
F9-OT9-R	GCCATCGTCATTAACAGGAGTG
F9-OT10-F	AGACAGACAGGAAGGCTATAGGA
F9-OT10-R	GTCATGCTGGGATTTAATCCC

Appendix Table S4 primer sequences for off target analysis of *in vivo* genome editing sgRNA of $F9^{Y381D}$ mutant mice.

ID	Sequence (5'-3')
$F9^{Y381D}$ -OT2-F	GTGGATACATGCATGTGAGTGG
$F9^{Y381D}$ -OT2-R	ACCGGTTCAGTTTCCTCTTCTG
$F9^{Y381D}$ -OT3-F	CAGGTGACAAGGAAGATGATGG
$F9^{Y381D}$ -OT3-R	CTCCAAGGAAACAAGAGGAACC
$F9^{Y381D}$ -OT4-F	CACTGGCAAAGATACTTCTGGG
$F9^{Y381D}$ -OT4-R	AAACCAGGGGAAAGGCAGTT
$F9^{Y381D}$ -OT5-F	GAGCAAATGTCTGTCTGCCAGA
$F9^{Y381D}$ -OT5-R	CCATTCTGAGGTCTTGTGACTGAG
$F9^{Y381D}$ -OT6-F	CTTATGGTCTTGAGCTGTACCAGC
$F9^{Y381D}$ -OT6-R	GAATGGGTTGTTCTTCTCACAGG
$F9^{Y381D}$ -OT7-F	GCTGTATCCTCTGGGTGTTTGT
$F9^{Y381D}$ -OT7-R	GGAGAGAAATAGGGTCAACAGC
$F9^{Y381D}$ -OT8-F	CACACCTGGCTTGAAAGGTT
$F9^{Y381D}$ -OT8-R	CAACTCTGGTCACCCTGACTTAA
$F9^{Y381D}$ -OT9-F	TTAGATGCACCCACTAGGCCTA
$F9^{Y381D}$ -OT9-R	GATTATTGAGTATGGCCCCAGG
$F9^{Y381D}$ -OT10-F	TTCAAACTCAGGAGCCTCCAGA
$F9^{Y381D}$ -OT10-R	GCTCATCCGCTGGTTCTAATGT

Appendix Table S5 primer sequences for sequencing and T7E1 assay.

ID	Sequence (5'-3')
F9-F1	TCGAACTATCCCTCATCACCAG
F9-R1	GTCTGTAAAGGGCATCACCCAT
F9-F2	GAACCCATAAAGCACTCTCAGC
F9-R2	GGCTGGACTTACAGAAATGGTG
F9-F3	CTTCCTCAAGTTTGGTTCTGGC
F9-R3	CTTCACACGAATCTTTGCCTCC

Appendix Table S6 primer sequences for QPCR.

**ID**	**Sequence (5'-3')**
IL6-F	GGGACTGATGCTGGTGACAA
IL6-R	ACAGGTCTGTTGGGAGTGGT
TNFα-F	AGAGCTACAAGAGGATCACCAGCAG
TNFα-R	TCAGATTTACGGGTCAACTTCACAT
IL10-F	GCTCTTACTGACTGGCATGAG
IL10-R	CGCAGCTCTAGGAGCATGTG
actin-F	CGTCATACTCCTGCTTGCTG
actin-R	GTACGCCAACACAGTGCTG
