# Supplementary figures and images for: CRISPR/Cas9‐mediated somatic correction of a novel coagulator factor IX gene mutation ameliorates hemophilia in mouse
Source: EMBO Mol Med. 2016 Mar 10;8(5):477–88. doi: 10.15252/emmm.201506039 (PMC5125832; doi:10.15252/emmm.201506039)

Appendix Figure S3B

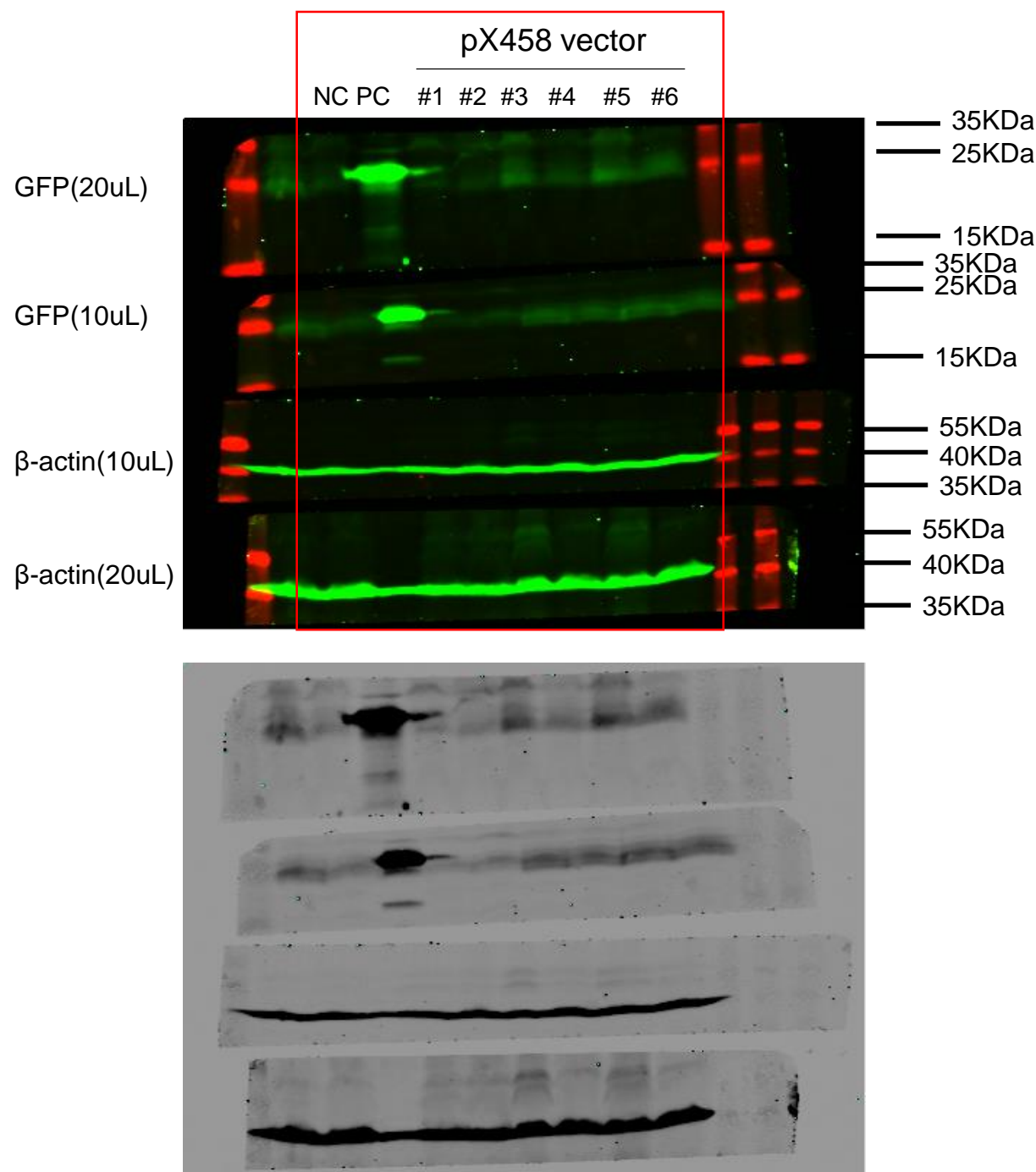

Supplement: Supplementary file 2 — Source Data for Appendix Figure [file EMMM-8-477-s002.zip › Source_data_for_Appendix/Source_dat_for_Appendix_Figure_S3.pdf]

Appendix Figure S2B

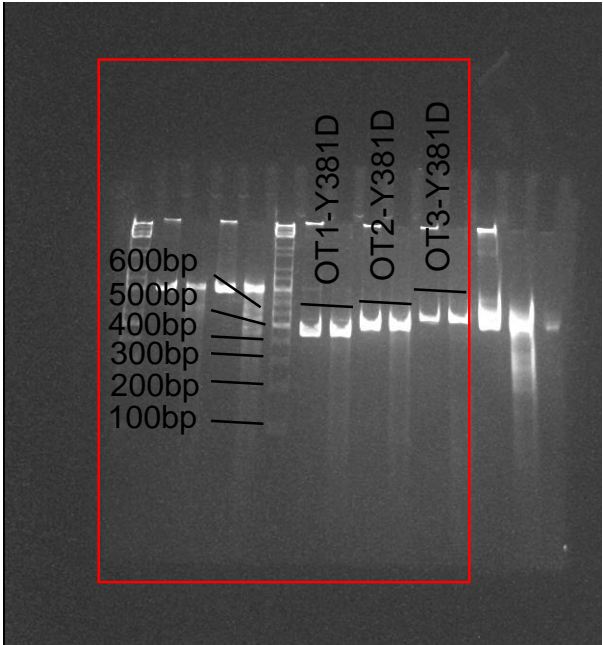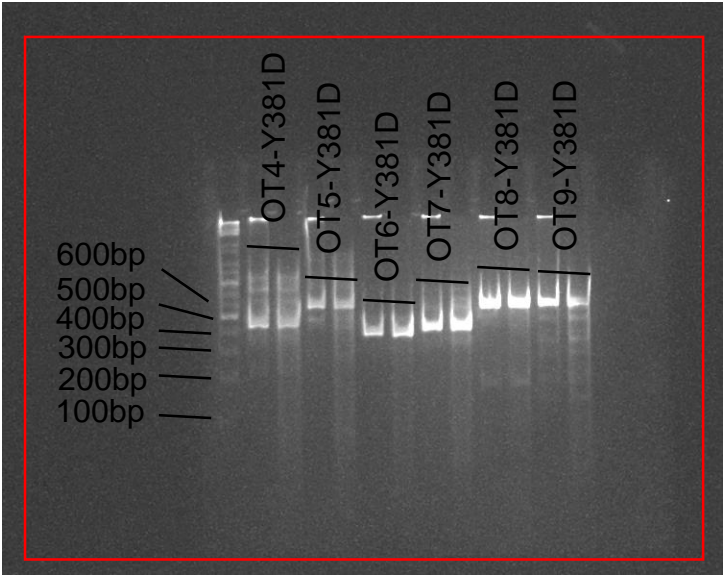

Appendix Figure S2B

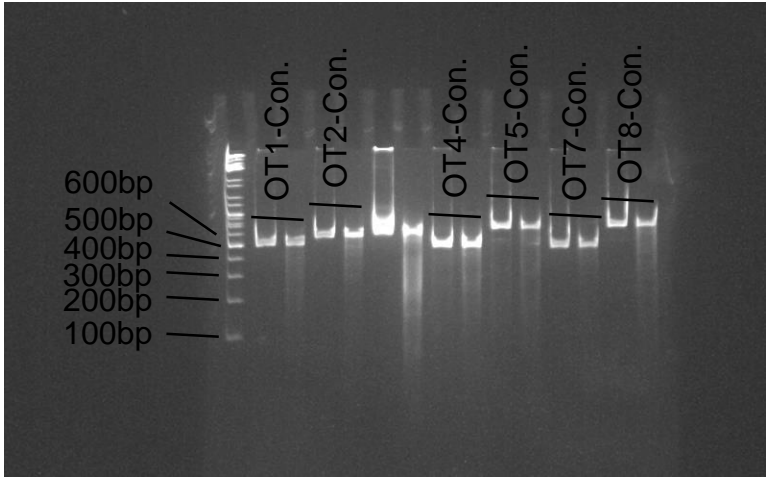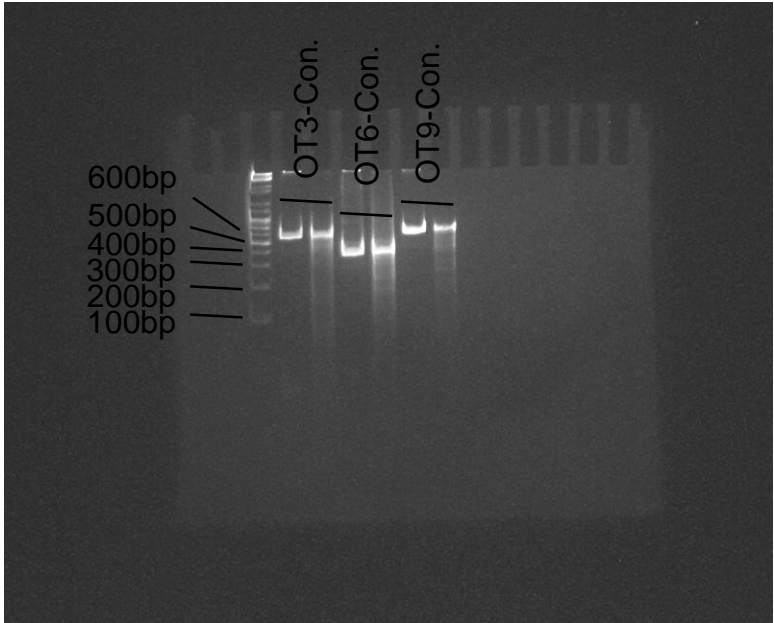

Supplement: Supplementary file 2 — Source Data for Appendix Figure [file EMMM-8-477-s002.zip › Source_data_for_Appendix/Source_data_for_Appendix_Figure_S2.pdf]

Appendix Figure S4B

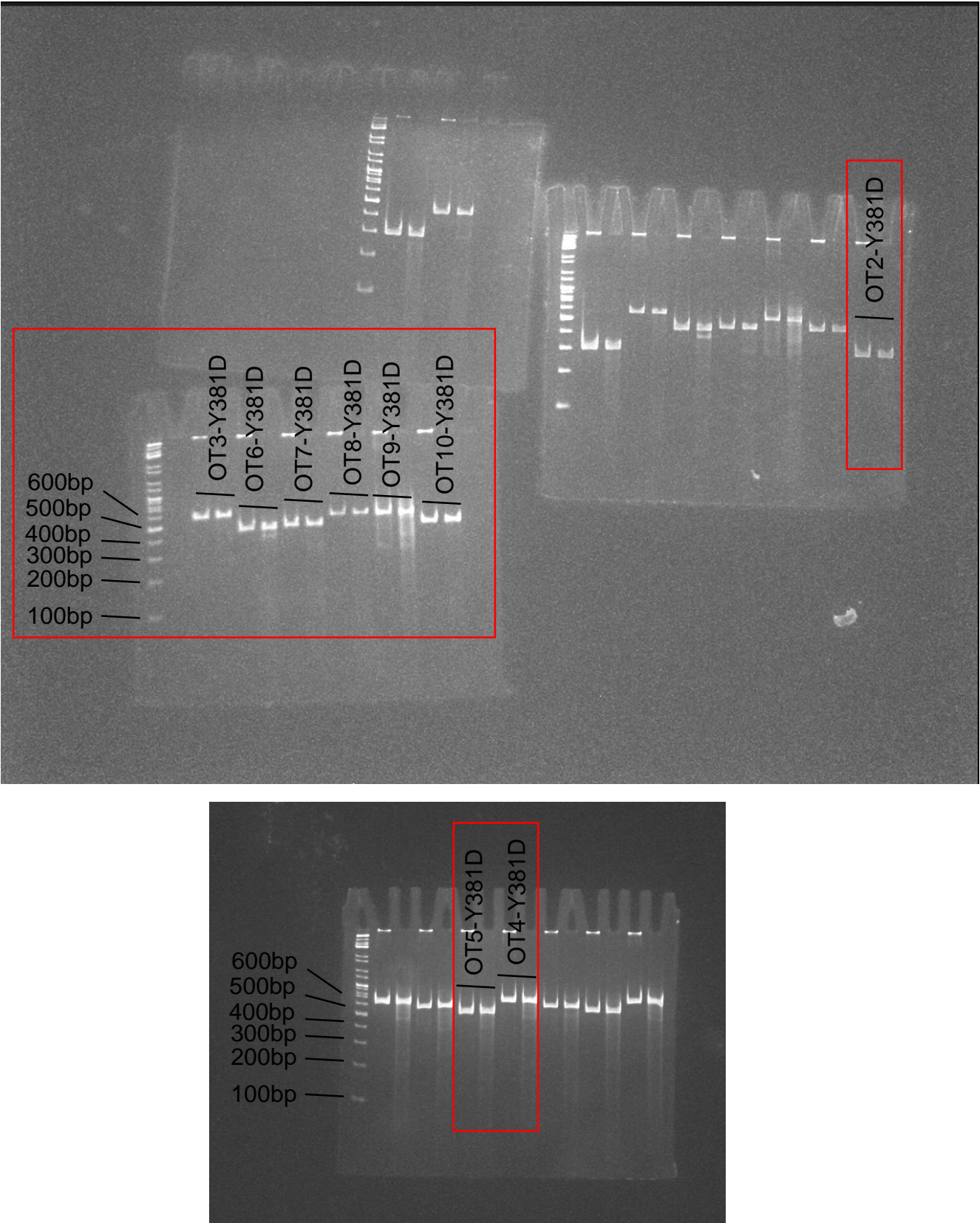

Appendix Figure S4B

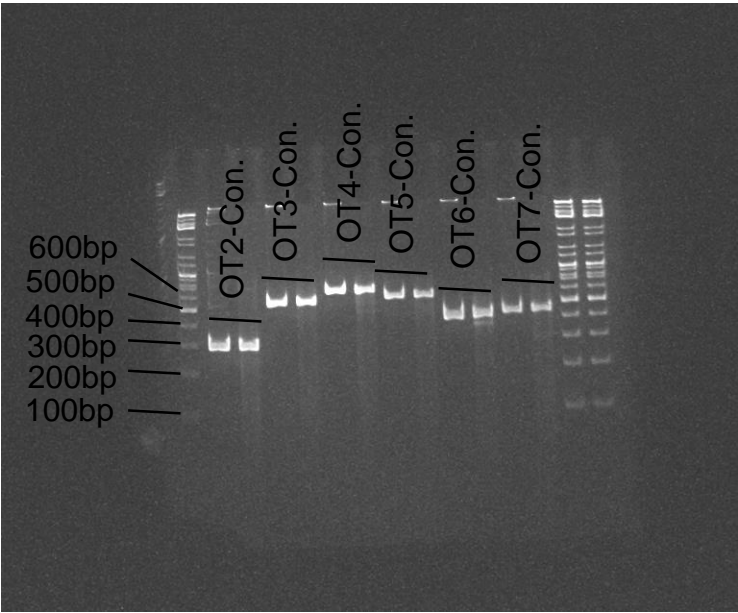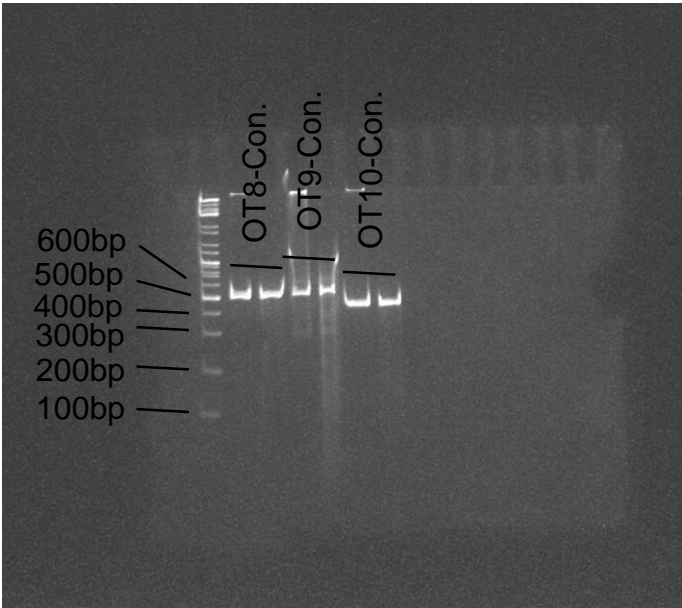

Supplement: Supplementary file 2 — Source Data for Appendix Figure [file EMMM-8-477-s002.zip › Source_data_for_Appendix/Source_data_for_Appendix_Figure_S4.pdf]

Figure 2C

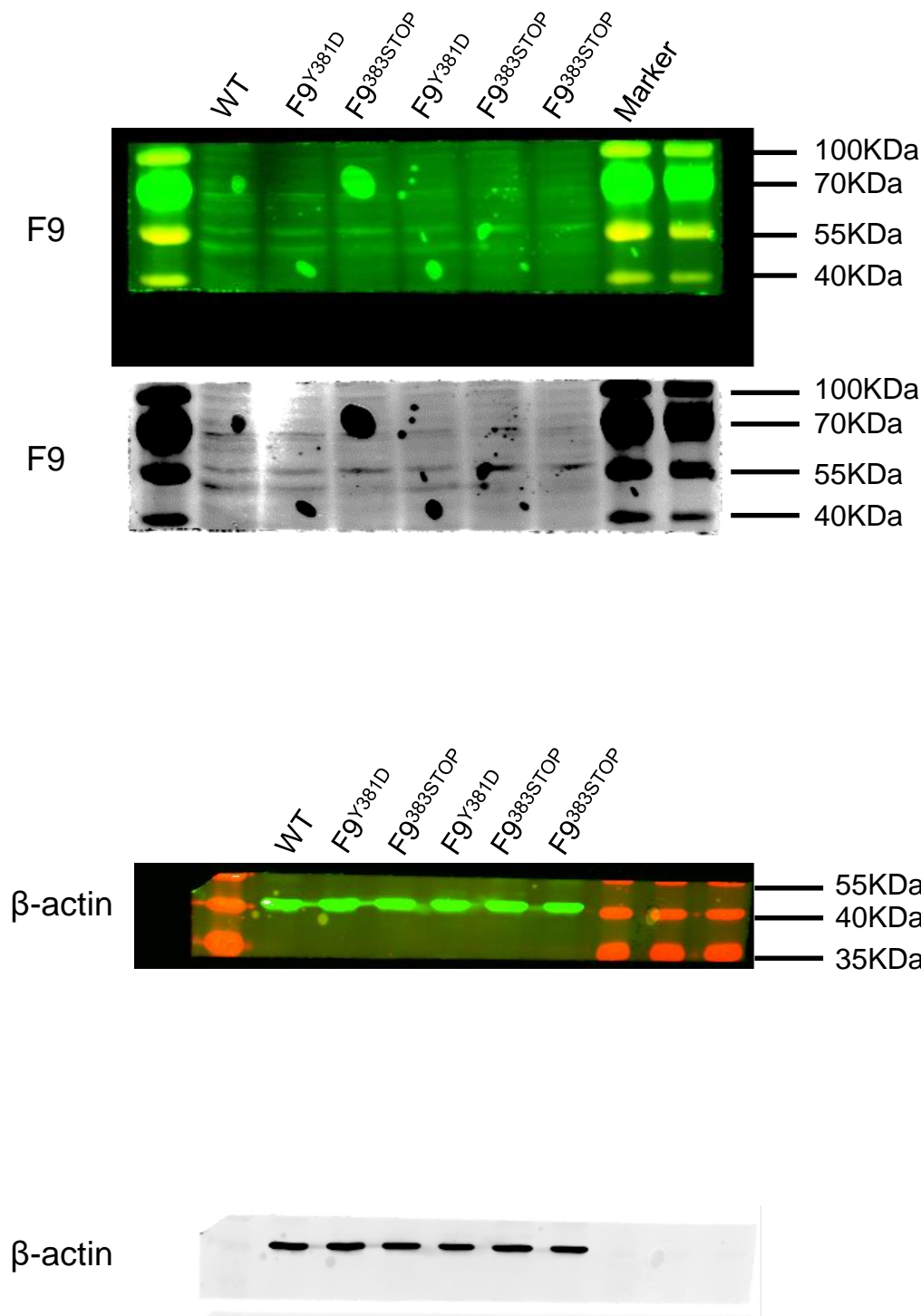

Supplement: Supplementary file 4 — Source Data for Figure 2 [file EMMM-8-477-s003.pdf]

Figure 4C

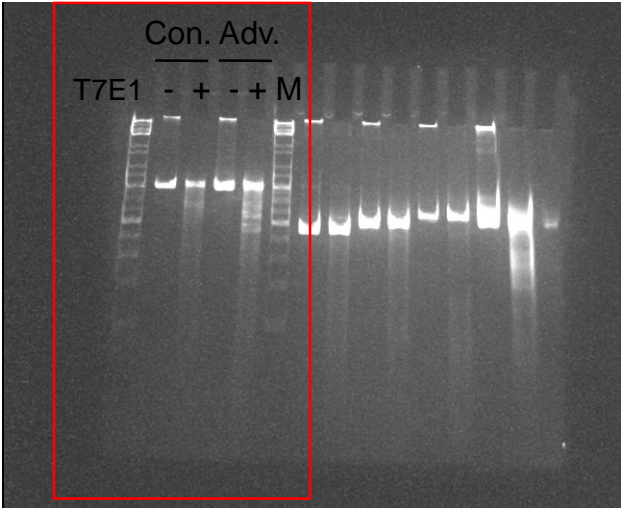

Supplement: Supplementary file 5 — Source Data for Figure 4 [file EMMM-8-477-s004.pdf]
